# Supplementary material for: Increased contact transmission of contemporary Human H5N1 compared to Bovine and Mountain Lion H5N1 in a hamster model
Source: Nat Commun. 2026 Mar 12;17:3869. doi: 10.1038/s41467-026-68900-8 (PMC13125286; doi:10.1038/s41467-026-68900-8)
Supplement: Supplementary file 1 — Supplementary information [file 41467_2026_68900_MOESM1_ESM.pdf]

Supplementary materials:

Increased contact transmission of contemporary Human H5N1 compared to Bovine and Mountain Lion H5N1 in a hamster model

This file includes:

1. Table S1. Summary of the amino acid variation among the three isolates
2. Table S2. Systemic and non-systemic virus spread in individual animals at 4 dpi.
3. Table S3: IHC analysis of virus antigen in tissues collected from individual animals at 4 dpi.
4. Table S4. Histology and immunohistochemistry analysis of respiratory tract tissues at 4 dpi.
5. Figure S1. Virus titers in oral swabs of donor animals during the transmission window.

Table S1. Summary of the amino acid variation among the three isolates

	PB2													
	58	353	362	441	489	607	615	627	631	649	663	667	676	682
A/bovine/Ohio/B24OSU-342/2024,Genotype B3.13 (EPI_ISL_19178076)	A	K	G	N	S	L	I	E	L	I	K	V	A	G
A/mountain lion/Montana/01/2024, Genotype B3.6 (EPI_ISL_19083124)	T	R	E	D	P	I	M	E	M	V	R	I	T	S
A/Texas/37/2024, Genotype B3.13 (EPI_ISL_19027114)	A	K	E	N	S	L	I	K	M	I	K	V	A	G

	PB1				PA										
	392	464	667	697	58	85	142	277	343	350	352	441	558	608	665
A/bovine/Ohio/B24OSU-342/2024,Genotype B3.13 (EPI_ISL_19178076)	V	D	I	E	S	T	K	S	S	S	D	M	S	T	L
A/mountain lion/Montana/01/2024, Genotype B3.6 (EPI_ISL_19083124)	I	D	T	G	G	A	K	P	A	N	E	V	L	S	L
A/Texas/37/2024, Genotype B3.13 (EPI_ISL_19027114)	I	N	I	E	G	A	E	S	S	S	D	M	S	T	M

	NP				NA		NS		
	52	105	230	433	382	389	7	40	85
A/bovine/Ohio/B24OSU-342/2024,Genotype B3.13 (EPI_ISL_19178076)	H	M	F	T	E	V	L	Q	P
A/mountain lion/Montana/01/2024, Genotype B3.6 (EPI_ISL_19083124)	Y	V	L	N	D	M	S	Q	S
A/Texas/37/2024, Genotype B3.13 (EPI_ISL_19027114)	H	M	F	T	E	V	L	R	P

	PB2-S1								PA-X			NS-1
	58	353	362	441	495	499	500	503	58	85	142	88
A/bovine/Ohio/B24OSU-342/2024,Genotype B3.13 (EPI_ISL_19178076)	A	K	G	N	I	R	S	D	S	T	K	T
A/mountain lion/Montana/01/2024, Genotype B3.6 (EPI_ISL_19083124)	T	R	E	D	V	Q	N	G	G	A	K	I
A/Texas/37/2024, Genotype B3.13 (EPI_ISL_19027114)	A	K	E	N	I	R	N	E	G	A	E	I

Table S2. Systemic and non-systemic virus spread in individual animals at 4 dpi.

Group	Bovine						Mountain Lion						Human					
Hamster no.	H1	H2	H3	H4	H5	H6	H7	H8	H9	H10	H11	H12	H13	H14	H15	H16	H17	H18
NT	2.6	2.97	3.27	8.8	7.75	8.25	5.6	4.66	3.6	4.6	4.05	3.71	0.5	7.64	5.57	7	4	3.6
Trachea	6.23	3.45	4.72	6.02	7.25	6.72	4.2	4.55	3.5	2.9	2.97	6.1	3.2	6.77	8.25	6.25	5.1	6
Lungs	4.5	0.5	6.75	3.75	8.75	7.97	4.5	2.67	2.6	0.5	0.5	0.5	3.75	6.95	6.8	4.02	6.3	3.9
Spleen	0.5	0.5	0.5	6.49	6.72	6.5	0.5	3.5	2.4	0.5	0.5	0.5	0.5	6.9	7.43	4.55	0.5	3.77
Liver	2.71	0.5	2.85	7.02	8.05	7.64	3.4	2.44	2.5	0.5	2.91	2.43	0.5	8.54	8.09	4.4	3.2	4.02
Kidney	0.5	0.5	3.28	3.02	5.19	5.39	2	2.92	0.5	0.5	0.5	0.5	0.5	5.32	5.57	3.65	2.9	3.27
Brain	0.5	0.5	0.5	7.26	7.11	4.86	2.7	2.59	0.5	2.6	0.5	0.5	0.5	5.85	5.62	4.08	2.5	2.6

Virus titers were quantified and are presented as log_{10} transformed values of $TCID_{50}/g$

The limit of detection was 0.5 log_{10} $TCID_{50}/g$.

Table S3: IHC analysis of virus antigen in tissues collected from individual animals at 4 dpi.

Group	Bovine						Mountain Lion						Human					
**Hamster number**	**H1**	**H2**	**H3**	**H4**	**H5**	**H6**	**H7**	**H8**	**H9**	**H10**	**H11**	**H12**	**H13**	**H14**	**H15**	**H16**	**H17**	**H18**
**Nasal turbinates**																		
Olfactory epithelium	2	0	0	4	4	4	1	0	0	0	2	0	2	4	3	0	1	1
Respiratory epithelium	2	0	0	4	4	4	0	0	0	0	0	1	0	4	3	2	0	0
Fibroblasts	0	1	0	3	3	3	0	1	0	0	0	0	0	3	2	0	0	0
**Trachea**																		
Macrophages, Epitheium	ntp	0	2	ntp	ntp	4	0	0	0	ntp	0	2	ntp	2	3	3	3	ntp
**Lung**																		
Macrophages, Pneumocytes	2	0	3	2	3	2	0	0	0	0	0	1	0	2	3	0	3	0
Bronchiolar epithelium	0	0	3	2	2	3	0	0	0	0	0	0	0	2	0	0	3	0
**Spleen**																		
Macrophages	2	2	2	4	4	4	0	3	2	1	1	1	2	3	4	2	1	2
**Liver**																		
Macrophages, bile ducts, hepatocytes	0	0	0	3	4	3	0	1	0	0	0	0	0	4	4	0	0	0
**Kidney**																		
	0	0	0	0	0	0	0	0	0	0	0	0	0	0	ntp	0	0	0
**Brain**																		
Olfactory, glial cells and neurons	0	0	0	2	4	3	0	0	0	0	0	0	0	3	3	0	0	0

IHC attachment
0=none
1=rare/few
2=scattered
3=moderate
4=numerous
5=diffuse

ntp: no tissue present

Table S4. Histology and immunohistochemistry analysis of respiratory tract tissues at 4 dpi.

**Histopathology**												
**Group**	**Bovine**				**Mountain Lion**				**Human**			
**Hamster number**	**H1**	**H2**	**H3**	**H4**	**H5**	**H6**	**H7**	**H8**	**H9**	**H10**	**H11**	**H12**
**Trachea**												
Epithelial degeneration and necrosis	3	3	3	1	0	0	1	3	2	3	4	1
Neutrophilic tracheitis	4	2	4	2	0	0	2	3	2	3	4	1
**Lungs**												
Necrosis and neutrophilic bronchiolitis	4	0	2	2	0	0	0	0	2	0	2	0
Interstitial pneumonia	2	0	1	0	0	0	0	0	0	0	1	1
Pulmonary edema	0	0	0	0	0	0	0	0	0	0	0	0
Alveolar leukocytes	2	0	2	1	0	0	0	0	1	0	0	0
**Turbinates**												
Respiratory epithelium degeneration and necrosis	0	0	0	0	0	0	0	0	1	1	0	0
Olfactory epithelium degeneration and necrosis	0	0	0	0	0	0	0	0	0	0	0	0
Infiltrates neutrophilic	0	0	0	0	0	1	0	0	1	2	0	0

0 = No lesions
1 = Minimal (1-10%)
2 = Mild (11-25%)
3 = Moderate (26-50%)
4 = Marked (51-75%)
5 = Severe (76-100%)

**Immunohistochemistry**												
**Hamster number**	**H1**	**H2**	**H3**	**H4**	**H5**	**H6**	**H7**	**H8**	**H9**	**H10**	**H11**	**H12**
**Trachea**												
Epithelium	2	2	2	2	0	0	0	3	1	2	4	0
Macrophages	1	1	1	0	0	0	0	1	0	0	1	0
**Cumulative score**	3	3	3	2	0	0	0	4	1	2	5	0
**Lungs**												
Alveolar macrophages	2	3	1	1	0	0	1	3	3	0	2	0
Bronchiolar epithelium	3	3	2	2	0	0	0	1	4	0	4	0
Pneumocytes	2	0	1	1	0	0	0	0	0	0	1	0
**Cumulative score**	7	6	4	4	0	0	1	4	7	0	7	0
**Nasal turbinates**												
Respiratory epithelium	0	1	0	0	0	3	0	1	4	3	0	2
Olfactory epithelium	0	0	0	0	0	2	0	0	1	0	0	3
Macrophages	1	1	0	0	0	1	1	0	1	1	0	1
**Cumulative score**	1	2	0	0	0	6	1	1	6	4	0	6

IHC attachment
0=none
1=rare/few
2=scattered
3=moderate
4=numerous
5=diffuse

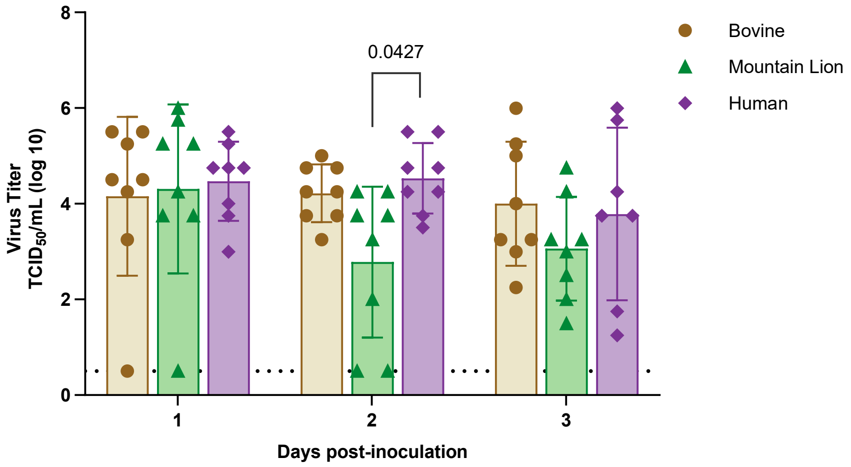

Figure S1. Virus titers in oral swabs of donor animals (n=8 animals per group) during the transmission window. Data are represented as the mean +/- standard deviation, with error bars indicating the standard deviation. Individual data points are shown. Significance was calculated using two-way ANOVA mixed-effects model followed by Tukey's multiple comparisons test. P-values adjusted for multiple comparisons < 0.05 are shown.
