# Supplementary material for: Increased contact transmission of contemporary Human H5N1 compared to Bovine and Mountain Lion H5N1 in a hamster model
Source: Nat Commun. 2026 Mar 12;17:3869. doi: 10.1038/s41467-026-68900-8 (PMC13125286; doi:10.1038/s41467-026-68900-8)
Supplement: Supplementary file 2 — Reporting summary [file 41467_2026_68900_MOESM2_ESM.pdf]

nature portfolio

Corresponding author(s): Dr. Vincent Munster

Last updated by author(s): Jan 14, 2026

# Reporting Summary

Nature Portfolio wishes to improve the reproducibility of the work that we publish. This form provides structure for consistency and transparency in reporting. For further information on Nature Portfolio policies, see our Editorial Policies and the Editorial Policy Checklist.

## Statistics

For all statistical analyses, confirm that the following items are present in the figure legend, table legend, main text, or Methods section.

| n/a | Confirmed | |
|---|---|---|
| ☐ | ☒ | The exact sample size (*n*) for each experimental group/condition, given as a discrete number and unit of measurement |
| ☐ | ☒ | A statement on whether measurements were taken from distinct samples or whether the same sample was measured repeatedly |
| ☐ | ☒ | The statistical test(s) used AND whether they are one- or two-sided<br>*Only common tests should be described solely by name; describe more complex techniques in the Methods section.* |
| ☒ | ☐ | A description of all covariates tested |
| ☐ | ☒ | A description of any assumptions or corrections, such as tests of normality and adjustment for multiple comparisons |
| ☐ | ☒ | A full description of the statistical parameters including central tendency (e.g. means) or other basic estimates (e.g. regression coefficient) AND variation (e.g. standard deviation) or associated estimates of uncertainty (e.g. confidence intervals) |
| ☐ | ☒ | For null hypothesis testing, the test statistic (e.g. *F*, *t*, *r*) with confidence intervals, effect sizes, degrees of freedom and *P* value noted<br>*Give P values as exact values whenever suitable.* |
| ☒ | ☐ | For Bayesian analysis, information on the choice of priors and Markov chain Monte Carlo settings |
| ☒ | ☐ | For hierarchical and complex designs, identification of the appropriate level for tests and full reporting of outcomes |
| ☒ | ☐ | Estimates of effect sizes (e.g. Cohen's *d*, Pearson's *r*), indicating how they were calculated |

*Our web collection on statistics for biologists contains articles on many of the points above.*

## Software and code

Policy information about availability of computer code

| | |
|---|---|
| Data collection | No software was used for data collection. |
| Data analysis | GraphPad Prism software, version 10.4.1<br>MAFFT for amino acid sequence alignment. |

For manuscripts utilizing custom algorithms or software that are central to the research but not yet described in published literature, software must be made available to editors and reviewers. We strongly encourage code deposition in a community repository (e.g. GitHub). See the Nature Portfolio guidelines for submitting code & software for further information.

## Data

Policy information about availability of data

All manuscripts must include a data availability statement. This statement should provide the following information, where applicable:

- Accession codes, unique identifiers, or web links for publicly available datasets
- A description of any restrictions on data availability
- For clinical datasets or third party data, please ensure that the statement adheres to our policy

Data included in this manuscript have been deposited in Figshare and publicly available. DOI: https://doi.org/10.6084/m9.figshare.30574997
A source data file is also provided.

## Research involving human participants, their data, or biological material

Policy information about studies with human participants or human data. See also policy information about sex, gender (identity/presentation), and sexual orientation and race, ethnicity and racism.

| | |
|---|---|
| Reporting on sex and gender | N/A |
| Reporting on race, ethnicity, or other socially relevant groupings | N/A |
| Population characteristics | N/A |
| Recruitment | N/A |
| Ethics oversight | N/A |

Note that full information on the approval of the study protocol must also be provided in the manuscript.

# Field-specific reporting

Please select the one below that is the best fit for your research. If you are not sure, read the appropriate sections before making your selection.

☒ Life sciences ☐ Behavioural & social sciences ☐ Ecological, evolutionary & environmental sciences

For a reference copy of the document with all sections, see nature.com/documents/nr-reporting-summary-flat.pdf

# Life sciences study design

All studies must disclose on these points even when the disclosure is negative.

| | |
|---|---|
| Sample size | The animal study sample size was determined by a power analysis based on data from previous literature.<br>For in vitro experiments, n=3 replicates were used to enable statistical testing. Exact sample sizes are provided in figure legends. |
| Data exclusions | No data was excluded from this study. |
| Replication | All animal experiments were performed once due to ethical considerations aimed at reducing the number of animals used. For in vitro experiments, n=3 replicates were used to enable statistical testing. |
| Randomization | Animals were allocated at random. |
| Blinding | Investigators were not blinded to group allocation during data collection or analysis, as the experimental interventions and outcome measures were objectively defined and did not permit practical blinding. The following analysis tasks were blinded: histopathology and immunohistochemistry analysis. |

# Reporting for specific materials, systems and methods

We require information from authors about some types of materials, experimental systems and methods used in many studies. Here, indicate whether each material, system or method listed is relevant to your study. If you are not sure if a list item applies to your research, read the appropriate section before selecting a response.

### Materials & experimental systems

| n/a | Involved in the study | |
|---|---|---|
| ☐ | ☒ | Antibodies |
| ☐ | ☒ | Eukaryotic cell lines |
| ☒ | ☐ | Palaeontology and archaeology |
| ☐ | ☒ | Animals and other organisms |
| ☒ | ☐ | Clinical data |
| ☒ | ☐ | Dual use research of concern |
| ☒ | ☐ | Plants |

### Methods

| n/a | Involved in the study | |
|---|---|---|
| ☒ | ☐ | ChIP-seq |
| ☒ | ☐ | Flow cytometry |
| ☒ | ☐ | MRI-based neuroimaging |

## Antibodies

| | |
|---|---|
| Antibodies used | Goat anti-hamster IgG antibody (Seracare Cat# 5220-0371), dilution of 1:2500 |

| | |
|---|---|
| Antibodies used | Anti-Influenza A nucleoprotein antibody (Millipore Sigma, Cat# ABF1820-25UL), 1:12,000 dilution<br>Rabbit IgG, Control Antibody (Vector Laboratories, Cat# I-1000-5), 1:2500 dilution |
| Validation | All of the above antibodies were validated by the manufacturer. |

## Eukaryotic cell lines

Policy information about cell lines and Sex and Gender in Research

| | |
|---|---|
| Cell line source(s) | MDCK cells were from Dr. Ron Fouchier, Erasmus Medical Centre, Netherlands, HTB cells from Cell Biologics (Cat# HM-6033). |
| Authentication | MDCK cell line was authenticated by Cytochrome b sequencing. HTB cells were not independently authenticated, as they were sourced from a commercial supplier. |
| Mycoplasma contamination | Cell line was tested regularly for Mycoplasma contamination. |
| Commonly misidentified lines<br>(See ICLAC register) | No commonly misidentified cell lines were used. |

## Animals and other research organisms

Policy information about studies involving animals; ARRIVE guidelines recommended for reporting animal research, and Sex and Gender in Research

| | |
|---|---|
| Laboratory animals | Syrian hamsters (Mesocricetus auratus) 4-6 weeks old (Envigo), mixed sex |
| Wild animals | N/A |
| Reporting on sex | No sex-based analysis was performed. |
| Field-collected samples | N/A |
| Ethics oversight | Rocky Mountain Laboratories Institutional Animal Care and Use Committee. Protocol number: 2024-029E |

Note that full information on the approval of the study protocol must also be provided in the manuscript.

## Plants

| | |
|---|---|
| Seed stocks | N/A |
| Novel plant genotypes | N/A |
| Authentication | N/A |
